# Socio-Economic-Political-Cultural Aspects in Malaria Control Programme Implementation in Southern India

**DOI:** 10.1155/2012/317908

**Published:** 2012-04-30

**Authors:** S. K. Ghosh, Rajan R. Patil, S. N. Tiwari

**Affiliations:** ^1^National Institute of Malaria Research (ICMR), Bangalore 560038, India; ^2^Community Health Cell, Bangalore, India; ^3^School of Public Health, SRM University Chennai, Potheri, Chennai 603203, India

## Abstract

*Objective*. A Socio-economic-political-cultural (SEPC) study was undertaken under the Roll Back Malaria (RBM) initiative to understand the process of programme implementation and how far in the changing malaria context, the broader environment has been understood and programme components have undergone changes. *Material and Methods*. Two studies were carried out; first in four villages under the primary health unit (PHU) Banavaralu in Tiptur Taluka in September 2002 and the second one in April 2003 in four villages in Chitradurga district, namely, Kappagere, Kellodu in Hosadurga Taluka, and Vani Vilas Puram and Kathrikenhally in Hiriyur Taluka. Focus group discussion and key interviews were adopted to collect the qualitative data. *Results*. Gender discrimination and lack of empowerment of women came out strongly in social analysis. In the rural elected bodies called *Panchayats*, the concept of health committees was not known. Health committees as one of the important statutory committees under every *Panchayat* were nonexistent in reality in these villages. Financial difficulties at *Grama Panchayat* level and also meager budget allocation for health have led to indifferent attitude of *Panchayat* members towards health. It was observed that there were generally no specific cultural practices in relation to malaria cure. Cultural and traditional practices in malaria-related issues were not predominant in the community except for some sporadic instances. *Conclusion and Recommendation*. SEPC study is an important indicator in malaria control programme. It is ultimately the community that takes the major decision directly or indirectly and the health authority must guide them in right direction.

## 1. Background

Vital endeavour of malaria control programme implementing activities requires first hand inventory of the community. In the existing health care delivery system, local stakeholders have not been adequately recognized [1]. Programme implementers have to realize that, in order to promote any health related activities, if the local actors are excluded, it is bound to fail. It is with this background this socio-economic-political-cultural (SEPC) study was undertaken under the Roll Back Malaria (RBM) initiative [2, 3] to understand the process of programme implementation, and how far, in the changing malaria context, the broader environment has been understood and programme component have undergone changes. 

Human resources at the community level often become serious bottleneck, which seriously interfere with the programme implementation and malaria programme is no exception. It would not be out of context to stress on the need for capacity building of the community is kept as priority when such national programme is being implemented. Though the emphasis continued to be on training and information flow to the programme implementation, reliable documented data on the available human resources both in the community and the trainers themselves are lacking [2, 3]. 

Apart from educating the community regarding malaria, there is a growing need to understand health seeking behaviour of the community [4]. It would just not be affordability but acceptance and compliance are equally important from the viewpoint of health service providers. 

It is needless to say that there is consensus about the need for RBM implementation. Concurrently, there is need for regular monitoring and evaluation of interventions under RBM. Since high-risk populations vulnerable to malaria are poor, there is a need to device propoor health system.

The key challenge before the health provider is to start working as a part of the health sector team with effective linkages with the like-minded departments/stakeholders [6, 7].

 Indicators to understand the constrains at the field level, which are essential for the community partnerships and maintenance of continued interest in the issue, have to be identified and prioritized for the effective implementation of the programme [5]. 

## 2. Study Villages

Two studies were carried out; first in four villages of PHU Banavaralu in Tiptur Taluka in September 2002 and the second one in April 2003 in four villages in Chitradurga district namely Kappagere, Kellodu in Hosadurga Taluka, and Vani Vilas Puram and Kathrikenhally in Hiriyur Taluka.

## 3. Methodology

This study was essentially an exploratory research carried out by a multidisciplinary team of researchers with background in epidemiology, sociology, and community development adopting qualitative methods of data collection to gain insights in to the community perspective and the provider perspective.

The community perspective was ascertained through a rapid social assessment of malaria-affected communities and application of standard qualitative techniques, namely, transect walk through villages, focused group discussion and in-depth interview. The **provider perspective** was obtained through key informant interviews with members of *Panachayat Raj Institutions*, and key officials and functionaries in health and other departments.

## 4. Findings

### 4.1. Social Issues

Gender discrimination is coming out strongly in all the villages against women in various walks of life. This applies from her childhood throughout women's life. Responding community amplified the discrimination—when they admitted differences were present right from admission of a girl child to school. Differences in agricultural wages were also another factor which undermined the position of women in the rural society. However, it should be noted that male members admitted that the contribution made by a women was at par with theirs. This economic disparity got reflected in the decision-making process of the family. The male decisions were predominantly accepted. This discrimination would reflect in health-related activities at family and community level.

In spite of women being elected to represent the community in *Grama Panchayat*, decisions are taken by their male representatives who are proxies in Panchayat meetings.

Subtle caste discrimination was also observed in study villages that could be barrier in effective implementation of malaria control programme for which total community participation is vital. Community knowledge on *Grama Sabhas* and health committees also has led to lower priority to health in *Grama Panchayat*.

Government policies have added to division in the community by giving more priority to underprivileged communities. Even in the distribution of below poverty line (BPL) card and various other development interventions instances were quoted where the noneligible families had benefited.

### 4.2. Economic Issues

Major occupation in the study area was agriculture and sericulture. Majority were BPL families having small pieces of dry tract of land. Treatment for illness for self and the family was unaffordable, due to high costs seeking treatment with private practitioners. The respondents agreed that the government hospitals (PHC) did provide medical services but they did not get satisfactory service from them, hence, they sought services of local general medical practitioners. As a result of high costs for medical treatment, many of the families are indebted to local moneylenders. Malaria has shattered economy of families in the villages. Every house has experienced malaria in the recent outbreak. People have incurred debts due to malaria. On an average, the treatment cost was in the range of 1500 to 12000 on malaria illness.

Due to drought and lack of other alternatives, families migrate in search of jobs outside their own village. There is a shift in agricultural practices from traditional crops to mulberry cultivation which is the main feed for silk worms. Since sericulture is an income generating semidomestic activity, the local farmers refuse to insecticide spray for toxic effects on the silk worms. 

The recent programmes initiated by the government seem to have not captured the imagination of the community in the field area. It was observed that the *Swachha Grameena* (clean village) programme had met with resistance, as community had to pay notional contribution for sanitation and general upkeep of the environment, which a had role to play in health situation of the community.

### 4.3. Political Issues

By decentralizing administrative powers through *Panchayat Raj Institution* (PRI), the government made a sincere effort to provide transparency, accountability, and social audit of various developmental programmes in the country. Karnataka state had taken lead by amending various acts pertaining to PRI to empower the rural community. Panchayat's role in public health activities is linked to the policies and directives of the health and rural development department. However, the *Panchayats* were empowered to take up various measures like environmental sanitation, and protected drinking water supply within limited resources that they were given. Because of limited resources and absence of administrative support, health activities had always remained in the backburner. This has resulted in apathy of elected Panchayat leaders towards health related programme to be taken up at the village level. It was found that the women representatives who had taken keen interest in health related activities expressed their ignorance and helplessness in voicing their concerns on public health in the *Panchayat* meetings at various levels.

*Grama Panchayats* feel they are not technically equipped to handle health activities. They also feel that health department staff should handle health, as Panchayat is politicized system and has got different priorities. This also reflects on poor intersectoral coordination in village development programmes. It was found many *Panchayat* members were either illiterates or neoliterates hindering their developmental thinking. As poor support from health department also adds to this situation, public health scenario (more particularly to malaria) in the area requires coordinated attention from all the concerned departments.

Community knowledge on *Grama Sabha* is found to be very poor and this had led to various development programmes ending as a failure in the villages. *Grama Sabha* is an effective platform for entire community to be a part of village development programmes is almost absent in every village, though it (conducting *Grama Sabha* twice a year) is mandatory according to Karnataka Panchayat Raj Act 1993. This absence has further led to deterioration of quality in development programmes, including health as there is no scope for community participation and community monitoring of programmes carried out by village *Panchayat*.

Concept of health committees is not known. Although health committee as one of the important statutory committees under every Panchayat is mandatory, according to Karnataka Panchayat Raj Act 1993, this is not existent in reality in these villages. This has led to lack of interdepartmental support to various health and development programmes at the village level. This has made public health a low priority for *Grama Panchayats*.

Financial difficulties at *Grama Panchayat* level and also meager budget allocation for health have led to indifferent attitude of *Panchayat* members towards health. Community feels most of funds to village *Panchayat* are programme based (like Swarna Jayanti Swarojgar Yojana (SJSY), Pradhana Mantri Gram Sadak Yojana (PMGSY), Indira Awas Yojana, Valmiki Ambedkar (VAMBE) Housing Yojana, etc.), and the same cannot be spent on general village development programmes.

### 4.4. Cultural Issues

It was observed that there were generally no specific cultural practices in relation to malaria cure. Cultural and traditional practices in Malaria-related issues were not predominant in the community except for some sporadic instances.

Local temple is visited to know whether the illness they are suffering needs to be attended at hospital or will it resolve by itself. This plays very important role as their treatment-seeking behaviour is influenced by this practice as people have profound belief in this activity.

*Tayeeta* (small copper or silver coin attached to a sacred thread usually put around shoulder or neck of a person that is believed to ward off evil) is collected from local priest during the illness.

## 5. Conclusion

SPEC study should be considered as an important indicator of malaria control programme. It is ultimately the community that takes the major decision directly of indirectly and the health authority responsibility is to guide them in right direction.

## References

[1] Narayan R, Sehgal PN, Shiva M, Nandy A, Abel R, Kaul S (1997). *Towards an Appropriate Malaria Control Strategy: Issues of Concern and Alternatives for Action*.

[2] WHO (2000). *Roll Back Malaria Guidelines for WHO-SEARO Region*.

[3] WHO (2000). *Implementation of Collaborative Activities on Roll Back Malaria in the South-East Asia Region: Report of an Intercountry Meeting*.

[4] Ghosh SK, Patil RR, Tiwari S, Dash AP (2006). A community-based health education programme for bio-environmental control of malaria through folk theatre (*Kalajatha*) in rural India. *Malaria Journal*.

[5] Sharma VP (1987). Community-based malaria control in India. *Parasitology Today*.

[6] National Vector Borne Disease Control Programme (NVBDCP) (2006). *Midterm Progressive Report Directorate of Health and Family Welfare Services*.

[7] Patil RR, Ghosh SK, Tiwari S (2011). Assessing perceptions about malaria among the elected representatives in rural India. *Tropical Parasitology*.

